# Building Techno-Scientific Trust in Adaptation to Sea-Level Rise in the Dutch Online Media

**DOI:** 10.1007/s00267-026-02620-z

**Published:** 2026-09-10

**Authors:** Ilona Bontenbal, Hanna-Mari Husu

**Affiliations:** LUT University, Lappeenranta, Finland

**Keywords:** Sea-level rise, Adaptation, Trust, Media, Techno-scientific trust, Technical solutions

## Abstract

In this article, we analyse how different adaptation strategies to sea-level rise (protection, accommodation and retreat) are presented and legitimised as approaches to climate-change resilience in the Dutch media. To examine how these strategies are positioned within media narratives, we draw on conceptualisations of trust, which help clarify why certain options gain greater legitimacy or credibility in public discourse. The analysis builds on three forms of trust: generalised trust, system trust and process-based trust. We find that Dutch media representations strongly legitimise technical and engineering-based protection measures, often at the expense of accommodation or retreat. Media narratives construct a clear hierarchy in which protection is most preferred and trusted, accommodation occupies a secondary position, and retreat is framed as least credible or desirable. This hierarchy is reinforced by depictions of substantial financial and institutional capacity to support large-scale technical solutions. Overall, the media reproduce trust in continuity and business-as-usual thinking, presenting futures that extend established socio-technical practices rather than imagining more transformative or disruptive adaptation pathways.

## Introduction

Sea-level rise poses a serious threat to many coastal areas in the world, including the Netherlands. Awareness and trust are often considered central factors to implementing effective climate change mitigation and adaptation (Hagen, Middel and Pijawka 2015; Kaltenborn, Krange and Tangeland 2017). This is also the case with implementing effective adaptation strategies to sea-level rise. Higher levels of social and institutional trust have particularly in the context of coastal management been related to greater community support for climate-related coastal policies (Jones and Clark 2013, 2014).

The Netherlands, as it is today, is the result of centuries of water and flood management practices (Olsthoorn 2008: 105), and the country has been considered a pioneer in testing different adaptation strategies to sea-level rise (Song and Peng 2017). Flood safety is ruled by national laws and implemented by national government organisations. An elaborate system of high dikes, dunes and sluices have been established to protect the country against sea floods. (Tol et al. 2006). Central aspects of the water management system include the massive Delta works that were initiated after the 1953 flooding disaster to shorten the shoreline, various storm surge barriers such as the Eastern Scheldt Barrier and the Maeslant Barrier, and the Afsluitdijk which separates the IJsselmeer from the Wadden Sea. Such large-scale projects illustrate that the Dutch system for water management has historically been founded on technological and engineering solutions and expertise, and the approach to flood risk management has predominantly been based on the notion to fight against water (Jong and Brink 2017; Roth et al. 2021). After the 1990s, there has been a paradigm shift from flood risk prevention towards a more nuanced approach to flood risk management, which emphasises nature-based water management, spatial planning and room for water to flow, and which recognizes that extreme events cannot be avoided entirely (Bartholomeus et al. 2023; Bosoni et al. 2021; Van Buuren et al. 2016; Jong and Brink 2017). However, path dependency and lack of participatory avenues have in practice meant that the most effective approach to flood risk management is still widely considered to be prevention through engineering expertise (Neuvel and Van Der Knaap 2010; Van Buuren et al. 2016). In relation to this, Dutch households’ risk perception and interest in participation in protection against flood risk has been deemed low. The majority of the population regard the local, provincial and national governments as primarily responsible for protection against flood risk, which has lead to high confidence that things are being looked after properly (Mol et al. 2020; Terpstra and Gutteling 2008). Consequently, increasing risk perception has been identified as an important priority (Terpstra and Gutteling 2008; Song and Peng 2017).

We seek to contribute to this discussion by analysing how the media, in this case the online presence of traditional media outlets, present adaptation to sea-level rise. Climate change mitigation and adaptation are frequently discussed in the media (Farbotko 2005; Hase et al. 2021; Dirikx and Gelders 2010; Schmidt, Ivanonva and Schäfer 2013; Vu et al. 2019), and news websites are a major source of public information about climate change (Schäfer 2015; Andı 2020). Also sea-level rise has received increasing attention in the media (Rick, Boykoff and Pielke 2011; Bontenbal et al. 2026). The way that the media represents various issues related to climate change can have a significant impact on how those issues are perceived, interpreted, trusted and legitimatised (Bolsen and Shapiro 2018; Schmidt et al. 2013). Previous research has examined how climate issues are portrayed both within individual countries (e.g. Ahchong and Dodds 2012) and across multiple national contexts (e.g., Hase et al. 2021; Schmidt et al. 2013). It has been found that the role that the media has in presenting information and interpretations is particularly important in covering issues with which people are not frequently confronted in their daily lives (Dirikx and Gelders 2010; Schäfer and Schlichting 2014; Schäfer 2015), such as sea-level rise. Media representations matter because they shape how the public and policymakers interpret climate impacts like sea-level rise, influencing which adaptation measures are seen as legitimate (van Aelst and Walgrave 2011; Bolsen and Shapiro 2018).

Despite this growing body of research, it has not yet been examined how Dutch media represent adaptation strategies to sea‑level rise or how these representations relate to different forms of trust. This constitutes a knowledge gap in both climate‑communication and adaptation‑governance scholarship. The present paper addresses this gap by analysing Dutch media coverage through the lens of generalized, system‑based and process‑based trust. In doing so, it offers a novel contribution to understanding how adaptation strategies are legitimised in a national context where sea‑level rise is a politically salient but experientially distant issue. This contribution is particularly relevant given that global reviews show that recommendations from coastal adaptation studies are often ignored, making it essential to understand public perceptions of climate change—perceptions largely shaped by news coverage (Schäfer 2015; Gibbs 2016; Andı 2020).

The article seeks to answer the following research questions: (1) How are different adoption strategies to sea level rise (protection, accommodation or retreat) presented and legitimized as approaches to climate change resilience? (2) What forms of trust are mobilised in media representations of different adaptation strategies? To analyse the place assigned to these adaptation strategies within Dutch media narratives, we draw on conceptualizations of trust, which help illuminate how certain strategies are accorded greater legitimacy or credibility in public discourse. The article is structured as follows: in the next section, we will discuss the different adaptation options to sea-level rise and the conceptual framework on trust. Subsequently, the methodology will be introduced, which includes the selection and analysis of the media articles and the description of the analysis stages. Thereafter, the analysis follows, divided into three main subsections: trust in protection, trust in accommodation and trust in retreat. In the end, we present a discussion about the findings and draw some concluding remarks.

## Conceptual Framework: Adaptation Measures to Sea-level Rise and Types of Trust in These Measures

### Adaptation Measures to Sea-level Rise

To make sense of the way that the media represent society’s resilience and ability to adapt to sea-level rise, we turn to conceptualizations of adaptation options to sea-level rise. To be able to cope with sea-level rise, various measures have been adapted around the world. Within the research field, these have traditionally been divided into protection, accommodation and retreat measures (“The PAR classification”) (see e.g., Zhu et al. 2010; Gibbs 2019).

**Protection** measures fundamentally try to stop rising sea levels from impacting coastal areas. Protection measures can be divided into hard measures that include various technological and engineering solutions, such as building seawalls, dikes, artificial reefs and sand barriers, and soft protection measures that include, for example, dune rehabilitation, sand nourishment and the restoration of coral reefs, salt marshes, mangroves and oyster beds (Lebbe et al. 2021; Powell et al. 2019; Roelvink et al. 2021). Particularly infrastructure protection responses tend to be favoured in many places, and they are used particularly frequently in densely populated areas such as coastal cities (IPCC 2019). Hard protection measures are, however, often very costly and sometimes even unaffordable (Hinkel et al. 2018; Molinaroli et al. 2019). Protection measures have also been considered to have various negative environmental long-term impacts and unfavourable outcomes on coastal ecosystems (Molinaroli et al. 2019; Gracia et al. 2018).

**Accommodation** measures include a variety of methods which, instead of trying to keep rising water away, try to accommodate it. This can be accomplished by, for example, building houses on poles, building floating houses, modifying existing buildings and adapting drainage systems. Generally, what combines these responses is that instead of building infrastructure they include a range of technology, architectural and urban planning responses. (Lebbe et al. 2021.)

**Retreat**, on the other hand, refers to adaptation in which certain coastal cities, neighbourhoods and infrastructure are relocated further from the coast (Lebbe et al. 2021; Rocle and Salles 2018). Retreat is often considered highly controversial politically and socially (Barnett and O’Neill 2012), and there is a large variety of legal, operational, social, cultural, psychological, and economic considerations and barriers related to retreat from certain areas (Siders 2019). Many scholars in the field, however, consider that retreat will be the only solution for some areas in the long run (e.g. Gibbs 2019; Gornitz et al. 2020; Griggs and Reguero 2021; Haasnoot et al. 2021; Lepesant 2024).

In the analysis, we will look at how trust in relation to these adaptation measures is presented in the media and how the media articles describe desirable adaptation measures to sea level rise for the Dutch society.

### Conceptualizing Trust

To explore how adaptation strategies to sea‑level rise are positioned in Dutch media coverage, we turn to conceptualizations of trust. Trust is a central aspect of social relationships and society, as it enables cooperation and collective action between different individuals and groups in unpredictable and uncertain conditions (Laurian 2009: 372). Various crises and risks can threaten the stability of social order. To maintain social order, trust has a central role, since it reduces complexity in cases where transition can be unpredictable and contingent (Luhmann 1979). In other words, in order to reduce the complexity of society, trust between individuals and within the system is central. As such, trust is related to promises that future events can be predictable and controllable, and that others can be relied on (see Lehtonen and de Carlo 2019; Rousseau et al. 1998).

Various scholars have identified different types of trust, and there is a wide variety in conceptualisations and typologies (Freitag and Traunmüller 2009; Hardin 2002; Lane 1998; Levi 2001; McKnight and Chervany 2000; Rousseau et al. 1998). There is, however, no consensus about the exact definition of trust or the specific different categories it should be divided into (Uslaner 2002). For our analysis, we build on Johnson and Grayson’s (1999) four-fold conceptualisation of trust. This conceptualization establishes four types of trust: generalised trust, system trust, process-based trust and personality-based trust (see e.g., Lindgreen 2003). In our analysis we, however, exclude the category of personality-based trust. We do this because the category of personality-based trust is based on individuals’ inherent propensity to trust, and thus it is not something to look for in the media reporting as it is closely tied to an individual’s personal traits.

**Generalised trust** is based on a general level of confidence that individuals maintain in the absence of reasons to doubt. Generalised trust grows out of how people imagine the future (for example in relation to sea-level rise), what they expect from others, and the promises that structure social cooperation. It is defined by shared norms of behaviour (Coleman 1988) and enforced by social mechanisms, such as peer pressure and the threat of ostracism (Johnson and Grayson 1999). People’s expectations shape how we anticipate others will behave, imaginaries provide shared visions of how society ought to work, and promises help stabilize those expectations, making trust possible even without enforcement. Generalised trust can relate, for example, to what kinds of technological and social orders people can expect. It is somewhat similar to what McKnight and Chervany (2000) define as situational normality, which entails trust that success is likely because the situation is normal or favourable. In other words, as long as there are no abnormal situations, people tend to trust others (Garfinkel 1963).

**System trust** is determined by a community’s written rules and on the effectiveness of regulatory institutions in enforcing these rules (Johnson and Grayson 1999). System trust is built on how people expect institutions, such as governments, technologies, markets or bureaucracies to behave, the imaginaries people share about how the systems work, and the promises that the institutions make. Expectations are thus forward-looking assumptions about how a system will behave and, as such, they are considered to reduce uncertainty and coordinate action by stabilizing what people think will happen (Arnaldi and Bianchi 2016). System trust is similar to what some other researchers define as institutional trust, which reflects the security that an individual feels about a situation because of guarantees, contracts, regulations, promises, legal recourse, processes, or procedures that are in place that assure success safety nets, or because of other organisational control structures (Shapiro 1987; Zucker 1986). In this type of trust, a key role is played by legislative and regulatory institutions, such as trade commissions and health departments (Johnson and Grayson 1999). Other institutions such as different types of organisations, public agencies, firms, and various scientific, parliamentary and democratic procedures, also play a role, as do scientific research and parliamentary and policy-making procedures (Lehtonen and de Carlo 2019: 3). As the degree of institutional trust is dependent on the institutions’ performance, perceptions related to equity, reliability, competence, and transparency are central (Levi and Stoker 2000; Lafuente et al. 2018). These elements shape whether people believe systems are reliable, legitimate, and worthy of trust.

**Process-based trust** is developed through repeated interactions between partners. Instead of being based on general social norms or the rule of law, it is based on individual expectations developed by two or more partners through repeated experience. This type of action depends on the behaviour of each partner and the history of interactions among the partners. Due to its essence, process-based trust is built through “spiral re-enforcement”, which means that this type of trust generally moves from being fragile to being resilient. (Johnson and Grayson 1999.) Rousseau et al (1998) refer to this type of trust as relational trust, which derives from repeated interactions over time between the trustor and the trustee. In this, the information available to the trustor from within the relationship itself forms the basis of relational trust. Process-based trust makes people confident that outcomes are trustworthy because the process itself is trustworthy, not because of personal relationships or institutional charisma. As such, process-based trust relies on the fairness, consistency, and reliability of procedures.

## Materials and Method

Three Dutch national online media sites were selected based on their significance (readership) and a balance of political leaning. These are (1) the news website of **the national broadcasting company (NOS)**, which is considered the second biggest online actor in the Netherlands (Kormelink & Lamot 2025), (2) the website of the largest tabloid paper, **De Telegraaf**, which is traditionally considered slightly more right-leaning, and (3) the website of the third largest newspaper, **de Volkskrant**, which is traditionally considered slightly more left-leaning (see e.g. Pew Research Centre 2018 on political alignment of Dutch news media). The Dutch media landscape is highly digitised, concentrated and pluralistic, dominated by public service broadcasting through the NPO (represented by NOS in our study) alongside major commercial publishers such as Mediahuis (represented by De Telegraaf in our study) and DPG Media (represented by De Volkskrant in our study).

**Fig. 1:**
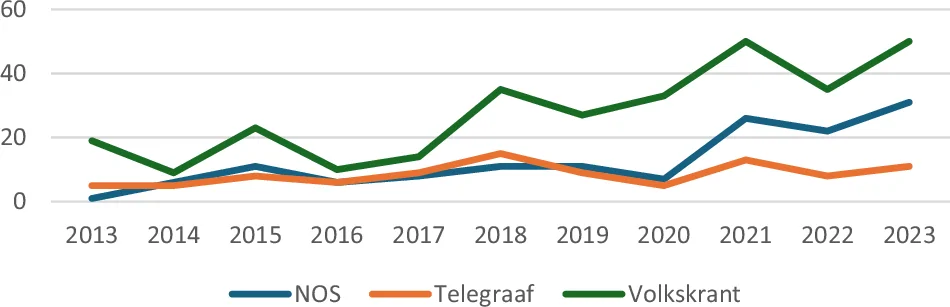
Number of articles mentioning sea level rise in the Dutch media

A keyword search was conducted in February 2024 on the official website of the selected media sites using the Dutch keyword “zeespiegelstijging”, which translates to “sea-level rise”, for a ten year time period of 1.1.2013–31.12.2023. A ten‑year period was selected for the analysis because it provides a sufficiently broad window to capture contemporary media representations of sea‑level rise without extending into older material that would shift the study toward a historical perspective. We decided to focus on media articles published online as in the Netherlands, major publishing houses have shifted to a majority-digital subscriber based. The online content of NOS has a 31% reach weekly of the population, the Telegraaf 16% and the Volkskrant 7%. (Kormelink & Lamot 2025). The analysis focused on text-based articles, and news videos were excluded. This keyword search produced a total of 539 articles (NOS: 140, Telegraaf: 94, Volkskrant: 305), all of which were included in the analysis. See Fig. 1 for the number of articles.

Thematic coding was conducted (by the first author using qualitative analysis software Nvivo) in line with Braun and Clarke’s thematic analysis guidelines (2012) to identify recurring patterns in how adaptation to sea‑level rise is discussed and to interpret the significance of those shared themes. First, we conducted a round of coding during which all mentions of the adaptation options, according to the three-fold typology of protection, accommodation and retreat, were coded. We thus looked for different adaptation descriptions in the media articles and coded them under the three-fold categorization. Under protection, all discussions and mentions of various hard and soft protection measures, such as strengthening dikes, building sea walls and adding sand to beaches were coded. Under accommodation, everything that was considered to indicate measures that aim to accommodate homes, neighbourhoods and societies to sea-level rise were coded. This included all mentions of raising houses, building floating houses, providing room for water to flow, making changes to infrastructure to be able to withstand rising waters, amending existing harbours, and changing building policies and laws. Under retreat, all mentions that discuss moving away from the coast individually, as a community or as a society were coded. Simultaneously, an Excel sheet was filled which had all the articles listed, in which it was noted which country each media article focused on, and written down specifically which adaptation options were discussed in each article. it was also determined whether sea-level rise was the main theme of the media articles or only mentioned as one theme among others.

In the second phase, the thematically organized text segments (under each adaptation option) were then further analysed from the perspective of trust. At this stage, the conceptual lens of different forms of trusts guided the analysis. Media texts that described broad societal expectations about how people and communities would respond to sea‑level‑rise adaptation, confidence in shared social norms and trust in the normal operation of socio‑technical orders, were categorised under **generalised trust**. Texts that emphasised confidence in formal institutions, regulatory frameworks and established socio-technical systems, including trust in governmental agencies, scientific procedures, legal guarantees, coastal protection technologies and the perceived competence, reliability or transparency of water-management authorities, were categorised under **system trust**. Texts that highlighted repeated interactions between actors involved in coastal management, or portrayed trust as emerging from established routines and accumulated experience with adaptation practices were categorised under **process-based trust**. The line between the different forms of trust was at times ambiguous, but the main difference is that generalised trust was understood to become visible in media coverage of adaptation to sea-level rise when articles rely on broad societal expectations rather than institutional assurances or procedural reliability.

The excerpts from the media used in the analysis are all translated from Dutch to English by the first author. In the analysis, also excerpts from interviewed experts are used. These quotations do inform us about the media’s framing because source selection is an editorial choice as well as the selection of which quotations to use in the final media article. However, it is important to note that the substantive views expressed by the interviewed experts reflect their professional judgement rather than the newspaper’s own position.

## Results: Presenting Trust in Adaptation Measures to Sea-level Rise

We found that there is an upward trend in the number of media articles published in the Dutch media that mention sea-level rise. Of the 539 analysed media articles, 215 discuss or mention some type of adaptation strategy or measure to be taken to adapt to sea-level rise. Adaptation strategies were discussed in 52% of the articles from De Telegraaf, 43% from the NOS and 35% from de Volkskrant. See Table 1 for emphasis on the different adaptation options. In the following subchapters, we will describe how different adaptation options are discussed by the media and how certain strategies are accorded greater trust or credibility. We focus particular on which measures are portrayed as suitable and preferable for the Netherlands.

**Table 1,:** Emphasis of adaptation options in the media

	NOS	Telegraaf	Volkskrant
**Adaptation discussed**	60 (43%)	49 (52%)	106 (35%)
Protection focus	31 (22%)	16 (17%)	64 (21%)
Accommodation focus	8 (6%)	8 (9%)	13 (4%)
Retreat focus	13 (9%)	7 (7%)	10 (3%)
Other focus^a^	8 (6%)	18 (19%)	20 (7%)
**Adaptation not discussed**	80 (57%)	45 (48%)	199 (65%)
Total	140	94	305

^a^This includes on-structural measures which are outside the PAR model (see Dedekorkut-Howes, Torabi, and Howes 2020), such as changes in legislation, changing political leadership, creating funding mechanisms to support poorer countries to adapt to sea level rise and expanding our international treaties to deal with sea level rise.

### Trust in Protection Measures

In the Dutch media, protection measures are discussed notably more than the other adaptation measures: of the 215 articles that discussed some adaptation strategy to sea-level rise, 111 discussed protection measures, most often dikes, seawalls, and dams. There is a **generalised level of trust** in protection measures. It is implied that if action is taken (on time), problems related to sea-level rise are technologically and engineeringly solvable, also in the future: “Technically it is probably possible to keep protecting the Netherlands from flooding up to 3 m of sea-level rise. For this we need to consider adding more sand and building heavier dikes - - ” (NOS 9.10.2023.) Trust in adaptation to sea-level rise is particularly often represented in relation to various hard protection strategies, especially in technological and infrastructural protection measures. Dikes, dams and seawalls are presented as the main and most conventional option to adapt to sea-level rise, in the future as well: “To truly protect the Netherlands against flooding, dikes will have to be raised and strengthened on a large scale by 2050” (De Telegraaf 31.7.2014). The articles often also represent protection measures as something that have a long and traditional history in the Netherlands: “The Dutch have armoured themselves against a substantive rise in the sea level with dikes and the Delta works” (de Volkskrant 2.12.2019). There is thus a high level of **process-based trust** presented that emphasises continuity and previous success. Trust is often associated with hard protection measures, particularly with maintaining the status quo, in other words preserving current land use and avoiding major societal changes.

In relation to this, trust in protection against sea-level rise in the Dutch media is represented as a common effort, taken on a macro scale and implemented by the state, rather than as an individual-level effort: “If you put a couple of Dutch people in an area with great risk of flooding, what will they do? Right, they will start building dikes. But what do Americans do? The ones that can afford it will put their houses on poles. The rest have bad luck.” (Dutch water expert Henk Ovink in de Volkskrant 9.9.2013.) Thus, various individual-level protection measures, such as homeowners building small protection walls around their properties, are not represented in the Dutch media as something that is a meaningful solution. Such individual barriers are, however, mentioned in Dutch news media as a solution used in less developed countries. De Volkskrant reports on adaptation to sea-level rise on the Marshal islands: “What immediately stands out are the many different types of sea defenses that residents have built behind their homes. - - Some walls are made of household waste and rusted car parts. Others are made of boulders, held together by nets.” (De Volkskrant 11.5.2018.) These types of descriptions stand in contrast to how adaptation to sea-level rise is described in the media, in the Dutch context. Such perceptions signal **system trust**, as Dutch media implicitly reinforce confidence in expert‑led, technologically sophisticated protection measures by juxtaposing them with informal, non‑expert practices elsewhere, thereby strengthening trust in Dutch technical expertise and the idea that climate adaptation should remain in the hands of professionals.

In relation to protection measures, the media also presents a high level of trust in available funds to pay for the expensive macro-level technological protection measures. The staggering costs of the technological and engineering solutions are frequently mentioned. However, the costs are narrated as something that the Dutch can afford, which exemplifies a level of **generalised trust** in society being able to handle things: “You can count 1 billion extra per one metre of sea-level rise per year. We are in a perfectly fine position to afford to pay that.” (Bas Jonkman, professor of integrated hydraulic engineering at TU Delft, in de Volkskrant 25.1.2021.) Some of the articles, however, do contrast this with the fact that, in other areas of the world, being able to afford very expensive adaptation strategies is not the case, which leaves people in an unequal position.

Furthermore, besides bringing up traditional hard protection measures, the media also brings up various bold, expensive and large-scale technological and engineering-based protection solutions, which are outside of the mainstream. Such ideas include the Northern European Enclosure Dam (NEED) (a mega-dam intended to close the North Sea and make it into a type of a lake), propositions to build new islands on the coast, and large pumps to empty rivers and/or pump sea water into deserts. Such protection measures can be considered technological imaginaries (Vicente and Dias-Trindade 2021: 710) for the future. As such, these ideas are not represented in the same way as other hard protection measures through process-based trust, since they are described as something that does not exist yet and has not been done before. Presentations of these solutions rely on trust in the idea that technology can ultimately solve issues related to sea-level rise: “Sjoerd Groeskamp once started counting it as a joke, but the new IPCC climate report brings his ‘delusion’ one step closer. Two dams, totalling 637 kilometres, will close off the North Sea.” (De Telegraaf 16.8.2021.) Some of these solutions would drastically change the landscape of the Netherlands and Europe. Interestingly, however, the societal impacts of these large adaptation measures, such as building a whole new coastline, are not discussed, which could indicate that these ideas are not considered serious solutions, at least not in the coming decades. The media narratives thus foreground the technical feasibility of ambitious engineering proposals while downplaying their disruptive societal implications, thereby aligning with established socio‑technical expectations and reinforcing confidence in expert‑led coastal management

Besides the above‑described hard protection measures, Dutch media coverage also frequently highlights various soft protection measures, particularly beach nourishment and dune rehabilitation. While hard protection is represented through process-based trust and described as having historical significance, soft protection on the other hand is described as the way of the future: “In the Netherlands, we are used to fighting against waxing water, with the Afsluitdijk as a symbol thereof, - - but it is time for a new course of action: working together with nature.” (Artist Daan Roosegaarde in de Volkskrant 16.10.2021.) The use of soft protection measures is thus described as something more up to date and innovative compared to old-fashioned hard protection.

A particularly high level of trust is placed on experts and engineers providing technological and infrastructural solutions. This is also made visible by the media constantly interviewing experts, particularly Dutch experts, i.e., experts affiliated with Dutch institutions and universities, and relying on them to explain what sea-level rise is and how to respond to it. Experts are quoted in almost every article that discusses adaptation to sea-level rise. These quotes are used, for example, to explain academic findings and to bring a national angle to international reports. Four types of Dutch experts that are frequently referred to in the media particularly stand out: (a) the Delta commissioners, (b) representatives of the Deltares institute, (c) representatives of The Royal Netherlands Meteorological Institute (KNMI), and (d) academic researchers affiliated at Dutch universities. This reflects that much of the public debate about sea-level rise in the Netherlands is shaped by the output of major national, and in many cases governmental, institutions. These institutions provide diverse scenarios about living with rising sea levels in the future. Such scenarios are frequently reported by the media. Therefore, these institutions are an important voice in the public discourse. The media do not operate in isolation from wider society. In terms of trust, the heavy use of expert voices in the media reporting reflects both **system-based trust** as well as a level of **generalised trust**, i.e., a level of confidence in experts that is maintained in the absence of reasons to doubt.

Furthermore, there is a crosscutting theme in the media articles according to which the Dutch are particularly good at water management and solving problems related to sea-level rise, which is considered a point of national pride. The following excerpt from the media illustrates this: “Delta expertise is a Dutch export product.” (de Volkskrant 21.1.2021) and “As a country, we are the best at managing sea-level rise” (De Telegraaf 22.8.2015, quoting the prime minister Mark Rutte). This can be interpreted in relation to **process-based trust**, which is based on the Dutch water managers building a name for themselves in water management for centuries (see also Voogt et al. 2021).

Although protection measures are commonly represented in terms of the various dimensions of trust, it should be noted that certain doubts and statements of mistrust are also present in the Dutch media. In general, the various presented protection measures are considered to have certain limits, after which they are no longer reliable. There is doubt particularly in the long-term feasibility of technological protection measures: “I believe that we will come a long way if we continue doing what we have been doing thus far: adding sand to the coast, raising dikes. But there is a limit. With a sea-level rise of one-and-a-half metres, we have to think about far-reaching solutions.” (Jeroen Aerts, professor of water and climate risk at the Vrije Universiteit Amsterdam, in de Volkskrant 25.10.2021.) Expert opinions on the limits of using hard protection are frequently cited, and critical perspectives are represented as part of **system-based trust** in adaptation. One of the media articles quotes Professor Van de Wal: “We do not like to hear it in the Netherlands, but adaptation (adaptation to climate change) is not enough. We will in term not make it through raising the dikes. You also need mitigation: the emissions need to go down as soon as possible.” (De Volkskrant 25.9.2019.) Although the technological feasibility is not questioned per se, doubt is placed on, for example, whether such broad dikes could be accommodated in terms of existing infrastructure, whether they will be too expensive to build and also whether they will be socially acceptable.

### Trust in Accommodation Measures

Of the 215 articles that discussed some adaptation strategy to sea-level rise, 29 discussed accommodation measures. Compared to the protection measures analysed above, accommodation measures are thus less often referred to in the media, but when they are, they are described as measures that are more progressive and that will lead to more considerable transformations in society in the future. Therefore, the presenting of accommodation measures does not fall back on process-based trust, as much of the previously described protection measures do, but instead **system-based trust** plays a more important role in the narrating of future visions for accommodation.

The accommodation measures presented in the media are often related to how accommodation and societal infrastructure will be arranged in the future once society is faced with sea-level rise. Accommodation measures are thus represented as future-oriented solutions that rely on various technological promises. Some of the media articles envision that, to accommodate sea-level rise, individual houses as well as whole communities are going to be built on floating islands. Such accommodation measures will, however, require various futuristic technological solutions and thus the presentation is based on trust in technoscientific solutions. These techno-social visions are presented as an important safety net in the long term, but not something to rely on in the short term: “In case it goes completely wrong with the climate, then we will always have the many floating cities - -. In that way there will be less shortage of houses in the Netherlands, more space, and we will just be bobbing around happily with the sea-level rise.” (De Volkskrant 25.6.2021.) Some of the media articles also present less technological or high-level engineering-based accommodation solutions, such as building houses on poles. This strategy is however described as something that is happening in other countries, such as the US and on small island nations, but it is not envisioned as something that strongly pertains to the Netherlands.

In relation to housing and infrastructure, the most frequently brought up accommodation measure relates to future planning. The media articles emphasise that to adapt to sea-level rise, plans for the future on where to build housing need to be accommodated, which illustrates the rise of spatial planning in water management. There are reoccurring articles that describe how new neighbourhoods, from now on, will need to be built on higher ground and no longer in low-lying areas, deep polders or too close to rivers. The following quotation exemplifies this aspect: “There is an enormous challenge. The Netherlands needs almost a million new apartments, and these have to come primary to the Randstad. ‘In low lying areas. But if you do that, you know that in the long term, you will have to take all kinds of measures’, says Veenstra ( = interviewed expert, government architect). ‘For example, the roads will need to be raised. That puts a loan on the future generation and that is not something we will want.’” (NOS 16.9.2023.) To accomplish this type of accommodation, trust operates at the **system level**. More specifically, it is associated with the laws and regulations that govern building practices.

In relation to spatial planning, one of the accommodation adaptation measures that is frequently mentioned in the media articles is setting aside more room in society for nature. The media articles present giving more space to water and letting, for example, rivers flow more widely as an important part of adaptation to sea-level rise. Maarten Kleinhans, professor of physical geography, cited in the media: “The Netherlands has already given extra land to let rivers flow wider and to be able to store water in times of flooding. That is working well. At least until now.” (NOS 6.10.2022.) On similar lines, but perhaps more progressive, is the idea brought up in some of the articles that to adapt to sea-level rise, the Dutch should “give areas back to the sea” and focus on protecting some core areas of the country: “On the map of the Netherlands, she ( = interviewed expert, prof. Marjolijn Haasnot) draws a thick line with a pen around the Randstad: from Vlaardingen to Amsterdam, and from the Hague to Alphen. ‘This is dike ring area 14’, she explains. A zone permeated by dikes. ‘You could, for example, say: here we will make a large defence. And in other areas, we will accommodate.’” (Professor Marjolijn Haasnot in de Volkskrant 8.12.2023.) The justifications of accommodating sea-level rise by letting some areas overflow can also be seen to indicate a level of distrust in the current system and in continuing to do what has always been done. In general, the articles, however, present trust that giving up large areas is an unlikely scenario. Instead, they emphasise the feasibility of the technological solutions described in the previous section.

Besides accommodation in infrastructure and environment, there are some articles that describe the need for mental accommodation and adaptation. This narrative emphasises that people tend to have (**too) high levels or generalised trust** in adaptation to sea-level rise. The slow-paced nature of sea-level rise is considered to give the country ample time to react, which in turn is believed to lead to high levels of generalised trust: “‘At some point, it could happen [having to give up land], but it is a very slow process for which we can prepare ourselves’, says Glas ( = interviewed expert, Delta commissioner). ‘In this regard, we will look at all options’”. (NOS 28.1.2023.) This has led to people not being aware of the risks or preparing for them. The media articles emphasise that people should be more prepared for flooding and that adaptation to rises in sea level should not be taken for granted or left to the experts entirely. Furthermore, people should not rely on existing solutions but keep on working to improve adaptation, as noted by one of the experts, Quirijn Lodder, government water safety advisor, interviewed for an article in De Telegraaf: “But we would not fall into panic but instead stay alert and identify risks on time and leap onto them. - - One thing is for sure: our work is never done. We will always have to control and adjust to keep our feet dry.” (De Telegraaf 21.1.2023.)

### Trust in Retreat

Of the 215 articles that discussed some adaptation strategy to sea-level rise, 30 discussed or mentioned retreat as a potential solution. Retreat is presented by the media rather as the last resort that will happen when other measures fail or are no longer feasible. In this hierarchical relation of the different adaptation strategies, protection is at the top, followed by accommodation, while retreat is the least preferred and trusted as an adaptation measure, as illustrated by the following excerpt containing a citation from interviewed expert Maarten Kleinhans, professor of physical geography: “‘Different options need to be considered. Higher dikes will also become wider and wider, something that is currently impossible in some places. Rivers could also be made wider, as well as putting cities on poles and constructing islands in the ocean. And then there is one more option that, at the moment, no one wants to think about: giving up the current coastline and finding a place higher up’” (NOS 9.2.2019.) Because of retreat not being considered preferable, it is also described as something that should be stopped from happening through implementing various protection and accommodation measures. Retreat is thus represented in relation to a high level of **generalised and process-based trust** that things will be handled in a way that enables people to stay put: “‘Or we will find a place higher up. But I am not sure that anyone is looking forward to that option’, according to Haasnoot [=academic expert interviewed in article]. She hopes that the tide can be turned around through removing all the obstacles to reduce CO2 emissions.” (NOS 4.10.2021.)

However, when other adaptation options are expected to fail, mobility is regarded as a viable long‑term option, and some degree of trust is associated with this scenario,as noted by an interviewed expert (Prof. Pier Vellinga): “‘With double dikes you are now safe, and you can extend living in the Randstad for a couple hundred years. You are buying time.’ But eventually, he says, ‘more people will have to move to higher laying eastern parts of the country’.” (NOS 25.10.2021.) When retreat is discussed, it is mostly considered to mean that people from the western side of the country will have to be relocated to the high-grounded eastern parts. Some of the articles also discuss the potential of migration abroad, mainly to other European countries. The media present a level of **generalised trust** that the option to retreat will be available to the Dutch if it will be needed. Most of the articles that consider the retreat of the Dutch towards other European countries represent this as an option without representing distrust or doubt that neighbouring countries would be willing to let the Dutch in.

In the media, retreat as an adaptation measure is often also being considered in relation to the possible need to give up parts of the country to the sea. This topic is regarded as highly controversial, as exemplified by the following quotation: “‘I understand that from now on my life will be in danger if I say this, but it is what has to be done: decrease the stock of cattle, put dikes in the cities and let the lowest parts of the country run under water. Otherwise, we are digging our own seaman’s grave,’ he ( = Maarten Kleinhans, professor of physical geography) says.” (De Volkskrant 8.12.2023.) In some media articles, it is expressed explicitly that people will have to be relocated, whereas in other articles it is something that is discussed implicitly in relation to giving up land or, for example, submerging islands (in the Dutch case, particularly the Wadden islands). The possibility of having to let certain areas flood or submerge can moreover be considered an indication of distrust in the ability of protection and accommodation measures to keep the integrity of the current coastline in the (far) future, rather than an indication of trust in retreat. In the media, no clear plans are presented with regard to relocation or the idea of “turning back”, as it is often conceptualised, nor are the societal- or individual-level implications of large-scale retreat discussed. It seems that the discussion is not that far along yet. Furthermore, it should be noted that there are also several articles that instil trust in the readers by presenting relocation as a very unlikely scenario: “The possibility that areas of the Netherlands will in this century be abandoned is small, even though they are laying below the sea level.” (De Volkskrant 2.9.2019.) Mobility is mostly represented as a real-time occurrence mainly for small island nations or people from poorer countries, such as Bangladesh, not for the Dutch themselves, at least yet.

The Dutch media also presents distrust that the various adaptation measures, particularly retreat, are being considered and discussed on time, on a societal level, to make important and much-needed decisions about them (see also Tol et al. 2006), as exemplified by the following excerpt: “‘As far as I ( = Caroline Katsman, senior lecturer in physical oceanography) am aware, little thought is given to considering long-term alternatives to coastal management, other than spouting sand onto beaches and raising dikes.’” (NOS 9.2.2019.) This illustrates **a lack of system trust**. The statement explicitly criticises the established, expert‑led coastal‑management system by suggesting that political and institutional actors continue to rely on familiar protection strategies rather than exploring more transformative options. Read through the lens of system trust, this indicates that confidence in existing socio‑technical arrangements may limit consideration of alternative adaptation pathways, reinforcing reliance on traditional measures even when long‑term planning would require broader strategic thinking. In particular, distrust can be detected in the ability of politicians to ensure sufficient long-term decision making and preparation that goes beyond the traditional protection measures. This also relates to distrust in whether the Dutch as a society are prepared to act and to retreat if and when sea-level rise happens faster than expected. Because of this, retreat as an option is, according to some of the articles, expected to eventually fail. The inability to discuss and plan for retreat is considered to be hindered by the strong national emphasis on technologically based protection measures, as exemplified by the following excerpt: “‘In the Netherlands, the perception is very much so that with accommodation we will solve the problems. This is a misunderstanding.’ Not that an accelerated sea-level rise in the coming decades will lead to great problems. But for the period after that, there is much uncertainty.” (Quotation from interviewed expert Roderik van de Wal in NOS 9.2.2019.)

### Comparisons Between the Different Media’s Approaches to Adaptation Options to Sea-level Rise

When comparing the different media’s representation of the adaptation options with each other, we find that there are some differences to be noted. De Telegraaf had the most variety in its coverage of the adaptation options: 17% of all the articles mentioning sea level rise discussed protection measures, 9% accommodation measures and 7% retreat measures. In de Volkskrant and NOS, the dominance of the protection framework was more prominent: it was discussed in 21% of articles in de Volkskrant and in 22% of articles by NOS. Retreat as an option was discussed least frequently (relatively) in de Volkskrant (in 3% of articles). Accommodation was discussed fairly evenly in the three different media 9% in De Telegraaf, 6% in NOS and 4% of the articles in de Volkskrant.

While the analysis has primarily approached the ten-year period as a single analytical interval, several observations can nevertheless be made about the longitudinal dimension of media coverage. First, the total number of articles discussing sea-level rise increases steadily over the decade, indicating growing public and journalistic attention. Second, we do not observe any substantive shift in how different adaptation strategies are emphasised over time: protection measures remain consistently dominant, while accommodation and retreat continue to receive comparatively limited attention. A more specific temporal pattern emerges in the discussion of mobility as an adaptation solution, which appears across all selected media primarily in 2015–2016, with no notable increase thereafter. Taken together, these observations suggest that, despite rising coverage of sea-level rise, the hierarchy of preferred adaptation strategies remains remarkably stable. Future research could examine this longitudinal dimension more systematically, for example by analysing whether shifts in political agendas, extreme events or scientific assessments lead to changes in how adaptation pathways are framed over time.

Thematically considering, in the NOS (34% of articles) and De Telegraaf (30% of articles), sea-level rise was slightly more often the main topic of the article, whereas in de Volkskrant sea-level rise was less the main focus (in 23% of articles) and more often mentioned among other issues, often related to climate change in general. In terms of representation, the three media all had similar a focus emphasising the importance of protection. The main difference was that, in De Telegraaf, sea-level rise was at times represented from a denial perspective, closely tied to general scepticism towards climate change. This denial narrative was detected in 14 of the articles analysed from De Telegraaf, which represents c. 15% of all the included articles from this media outlet. From a trust-framework perspective, this pattern can be understood as distrust in the problem itself rather than distrust in specific adaptation strategies. In other words, these articles questioned the credibility of the underlying scientific assessments of sea-level rise, which positions denial as a form of **system-based distrust** rather than a lack of trust in particular solutions. This type of distrust rejects the need for adaptation, making particularly process-based trust irrelevant. Recognising this distinction adds an analytically productive dimension to the trust framework, as it highlights how some media narratives challenge the premise of adaptation rather than the strategies proposed. A denial perspective to sea-level rise was not detected in the other analysed media. The articles in the NOS were often shorter and more matter of fact, whereas the articles in de Volkskrant were longer and often contained interviews with experts. Both De Telegraaf and de Volkskrant also covered more issues related to techno-scientific imaginaries, such as the NEED mega-dam. The NOS had a smaller number of these more innovative solutions presented.

## Discussion—What do the Media Representations tell us About Desired Adaptation Solutions

In this article, we have examined how the media represent society’s resilience and capacity to adapt to sea-level rise. We analyse how different adaptation strategies (protection, accommodation and retreat) are portrayed across three Dutch traditional media outlets’ online content (NOS, de Telegraad and de Volkskrant). Our findings contribute to research on trust, resilience, and climate adaptation governance (e.g., Bord et al. 2000; Cologna and Siegrist 2020) by highlighting the potential media’s role in shaping perceptions of adaptation options and influencing policy and funding priorities. The paper contributes several conceptual insights into how trust shapes and is shaped by media representations of climate‑adaptation strategies:

We find that Dutch media representations largely legitimise technical–engineering solutions while sidelining alternatives such as accommodation or retreat (see also Olsthoorn 2008). Across the dataset, *system trust*, meaning confidence in expert institutions, engineering expertise and state‑led water management, acts as the primary stabilising force shaping how adaptation is imagined. Sea-level rise is presented as a technical problem requiring a technical fix, supported by strong system-based trust in engineering expertise and hard protection measures (see Song and Peng 2017; Joly and Le Renard 2021). Although Dutch water governance has formally shifted from prevention to risk management, our findings align with research showing that prevention remains the dominant and most trusted approach (Neuvel and Van Der Knaap 2010; Van Buuren et al. 2016). Such trust-building representations also justify substantial investment in technical solutions (cf. van Lente 1993). Even when high costs are highlighted, large-scale engineering projects are presented as feasible, which contrasts with depictions of less developed countries, where small-scale protection or retreat is presented as appropriate for “them” but not for “us”, reinforcing a narrative of Dutch exceptionalism and control and representing strong level of *generalized trust*.

Such a technosolutionist orientation, as Morozov (2013) argues, depoliticises sea-level-rise debates by translating political questions into engineering problems. This resonates with Roth et al. (2021), who show that the Dutch water sector remains anchored in a technical, conflict-averse discourse, reinforced by path-dependent institutional arrangements (Van Buuren et al. 2016). Media narratives frequently draw on *process‑based trust*, emphasising centuries of Dutch water‑management success. This form of trust frames protection as the natural continuation of what has always worked. Our findings suggest a mutually reinforcing dynamic in which this long-standing policy emphasis on engineering-based protection shapes how sea-level rise is reported, while media coverage that foregrounds technical solutions helps sustain imaginaries of continuity and “business as usual.” In this framing, adaptation possibilities become tied to techno-engineering logics rather than more imaginative or experiential pathways, and the challenges associated with sea-level rise are partially displaced from the political arena and recast as technical matters for experts.

At the same time, it is important to consider a plausible counterargument: media may not be narrowing public debate but simply mirroring an existing policy landscape that is itself strongly oriented toward engineering solutions. As noted by Jack (2020, 327) the media cannot stray too far away from accepted discourse if they want to be recognised and engaged with by their audiences. Because a content analysis cannot demonstrate audience effects or trace feedback loops between coverage, public opinion and policy, we present this interpretation as a theoretically grounded hypothesis rather than a causal claim. This positioning aligns with work on trust in socio-technical systems (cf. McKnight and Chervany 2000; Ketonen-Oksi and Vigren 2024) and clarifies how policy orientations and media narratives may co-evolve to shape the imaginative boundaries within which adaptation pathways are publicly discussed and acted upon.

It is also important to recognise that protection’s dominance in media coverage cannot be explained solely by a lack of trust in retreat. Because retreat is effective in reducing physical risk, distrust in its technical feasibility is unlikely to be the main issue. Instead, media tend to downplay retreat to avoid challenging widely accepted views, economic interests or political sensitivities. This emphasis on technological protection aligns with dominant public sentiment and ultimately reinforces the technocratic water‑management model that shapes Dutch coastal governance.

Throughout the media articles accommodation strategies appear mainly through futuristic techno-scientific visions such as floating communities, indicating some media role in imagining transformative futures. However, these representations are far less common than protection-oriented narratives and rarely address changes in social practices. Retreat is mentioned and trusted even less, though not entirely excluded as a long-term possibility.

While media narratives are not the sole determinant of public risk perception, they form a crucial part of the discursive environment in which adaptation decisions are made. Practical future pathways and recommendations on how to proceed for journalists and policymakers include that the public communication of adaptation to sea-level rise could be broadened beyond protection-centric narratives. Because media strongly legitimise engineering-based protection, communication strategies could intentionally diversify the portrayal of accommodation and retreat—not to advocate retreat, but to ensure that citizens understand the full spectrum of adaptation options and their trade-offs. As strong process-based trust in continuity and “normality” can foster complacency, societal actors should also communicate more clearly about uncertainties, limits and long-term risks associated with protection-only approaches. Journalists, for example, could adopt guidelines for reporting on uncertainty in coastal protection. Such practices would help bring adaptation choices back into democratic debate rather than leaving them solely to experts and would broaden who participates in imagining and shaping future adaptation pathways.

A key limitation of this study is its reliance on the single search term “sea‑level rise”, which may have excluded articles discussing adaptation under related terms such as “coastal risks” or “flood protection.” In terms of the sample, this means that the dataset is likely skewed toward articles in which sea‑level rise is explicitly foregrounded, potentially missing coverage embedded within broader climate, infrastructure or spatial‑planning debates. Future research could broaden the search strategy to include a wider set of terms capturing coastal risk and adaptation governance, and could also examine adaptation strategies beyond the PAR model, including land reclamation, institutional measures and other forms of planned intervention (Dedekorkut‑Howes et al. 2020). As our research is based on the web outlets of two national newspapers and the news platform of the public broadcaster, our findings represent only a certain segment of the Dutch media landscape. Future research could broaden this by looking also at the traditional print press, broadcast television and radio, as well as various online platforms and social media.

## Conclusion

Based on our analysis of 539 articles from the Dutch media from the time period of 2013–2023, we find that adaptation to sea-level rise is presented in the online media in a hierarchical relationship between the different adaptation strategies, in which protection is at the top, followed by accommodation, while retreat is least preferred and trusted as an adaptation measure. The Dutch media particularly legitimises technical engineering-based protection measures. Trust in such measures is built through process-based trust that relies on continuity in which future-making reproduces situational normality. We argue that such media representation presents a self-feeding loop in which the current approach in the Netherlands, which emphasises mainly technological solutions to rising sea levels, influences the way of media reporting. Simultaneously, the way of reporting which puts strong emphasis on technical engineering-based protection measures constructs a future in which possible and desirable futures are considered in relation to techno-engineering-based solutions. This approach enables people to remain to some extent distant to the challenges ahead, as the challenges become relocated from the political sphere and denoted the status of technological issues that are mainly seen as the problem of engineers and experts.

## Supplementary information

List of all articles included

## Data Availability

No datasets were generated or analysed during the current study.
